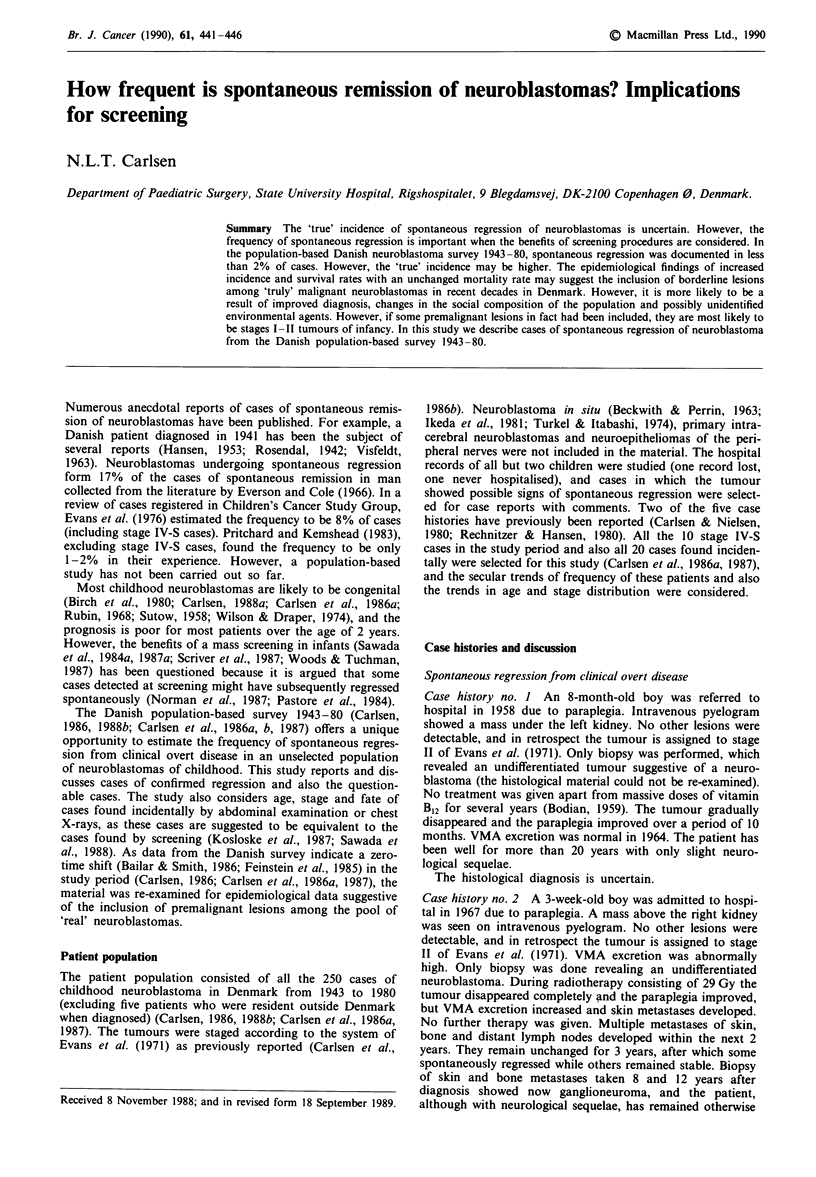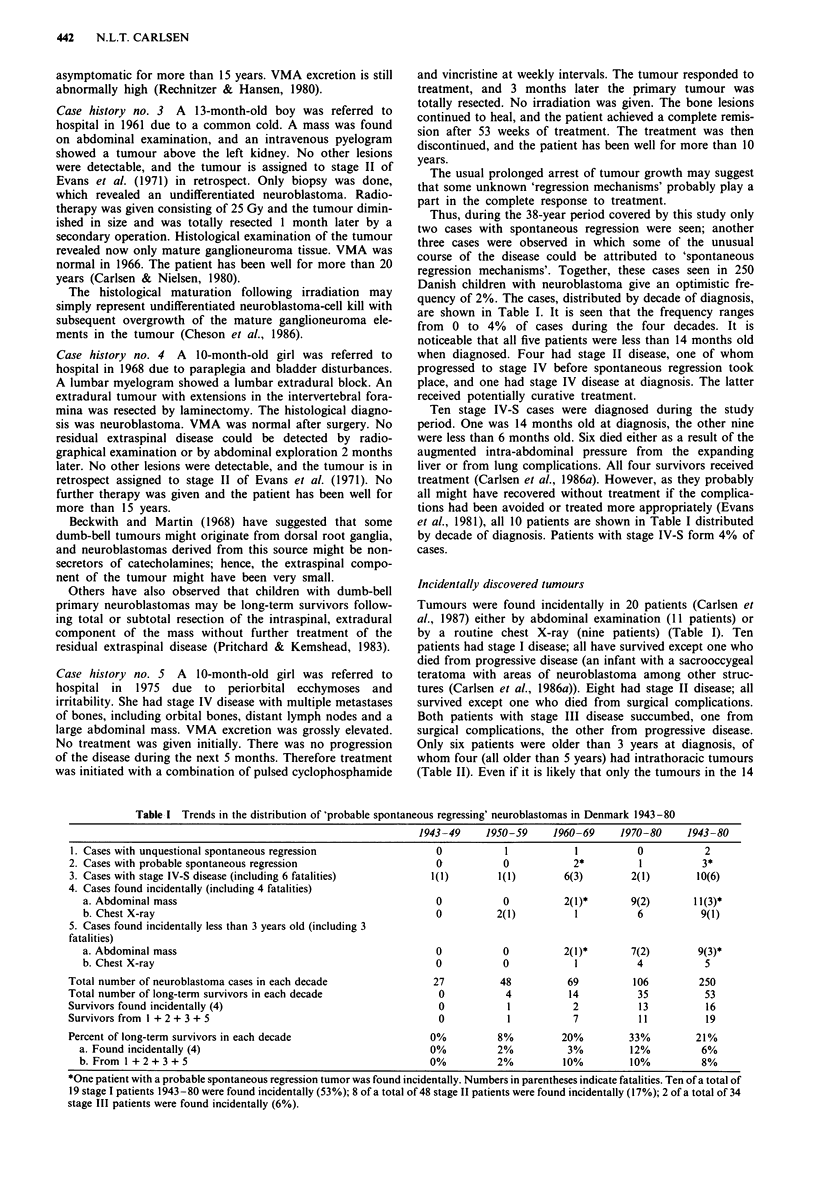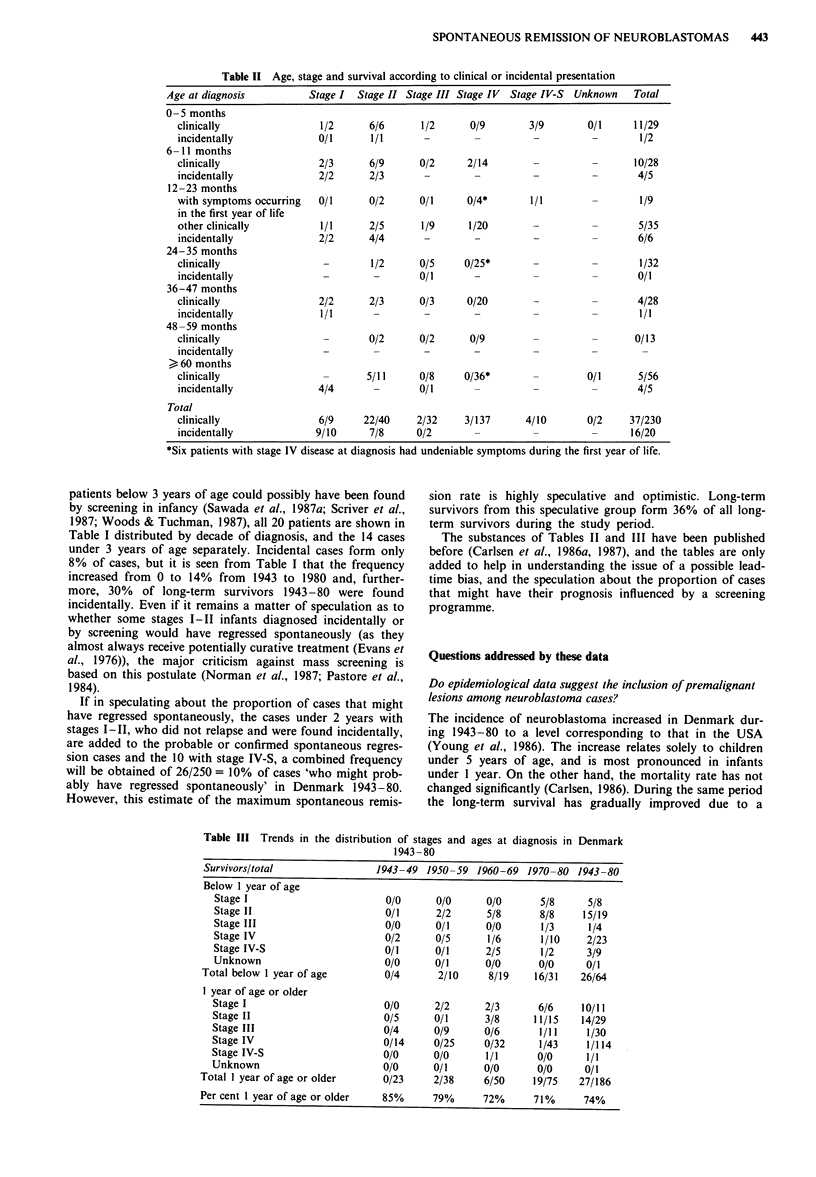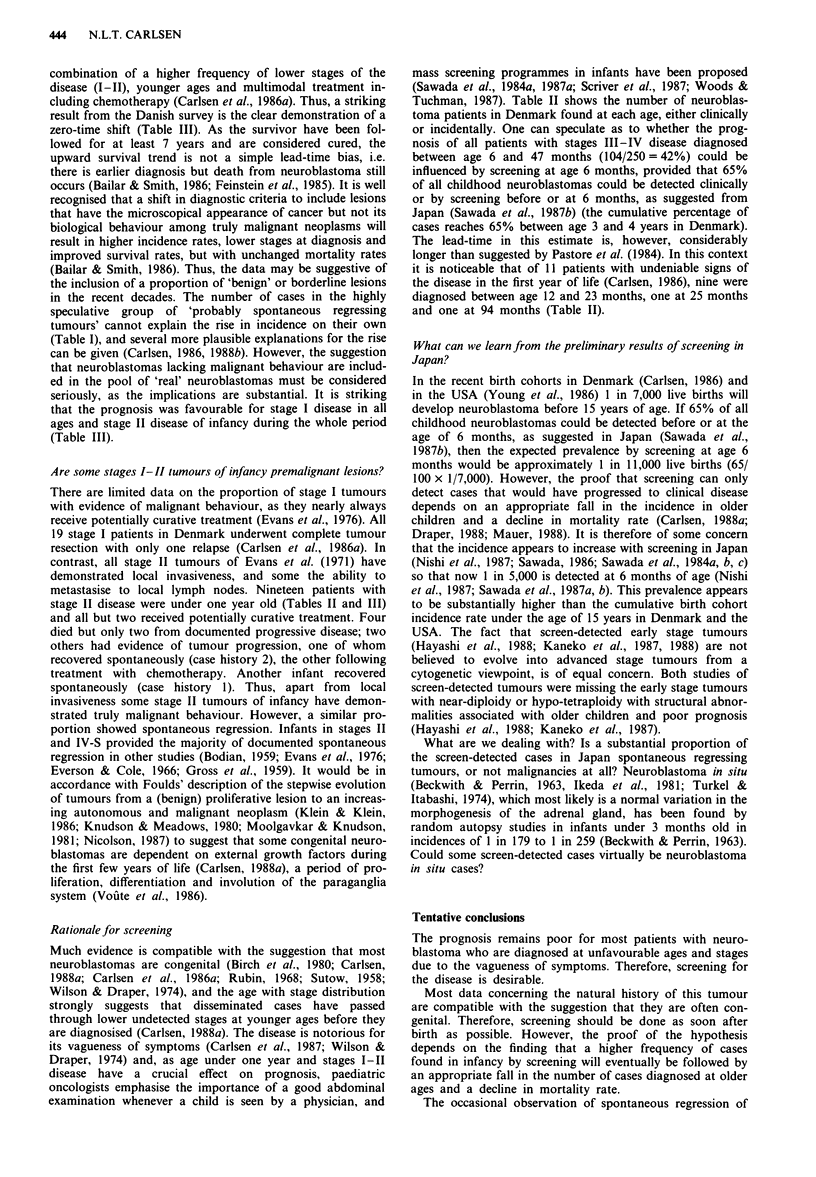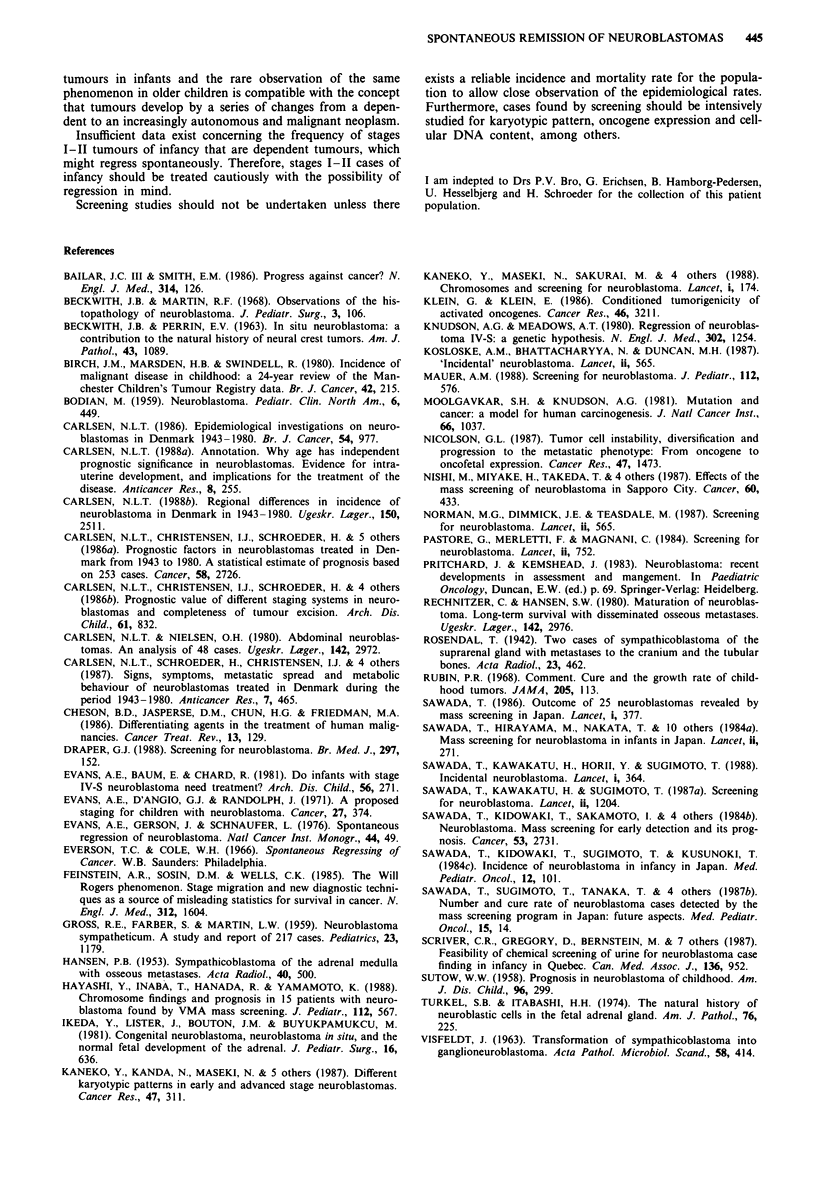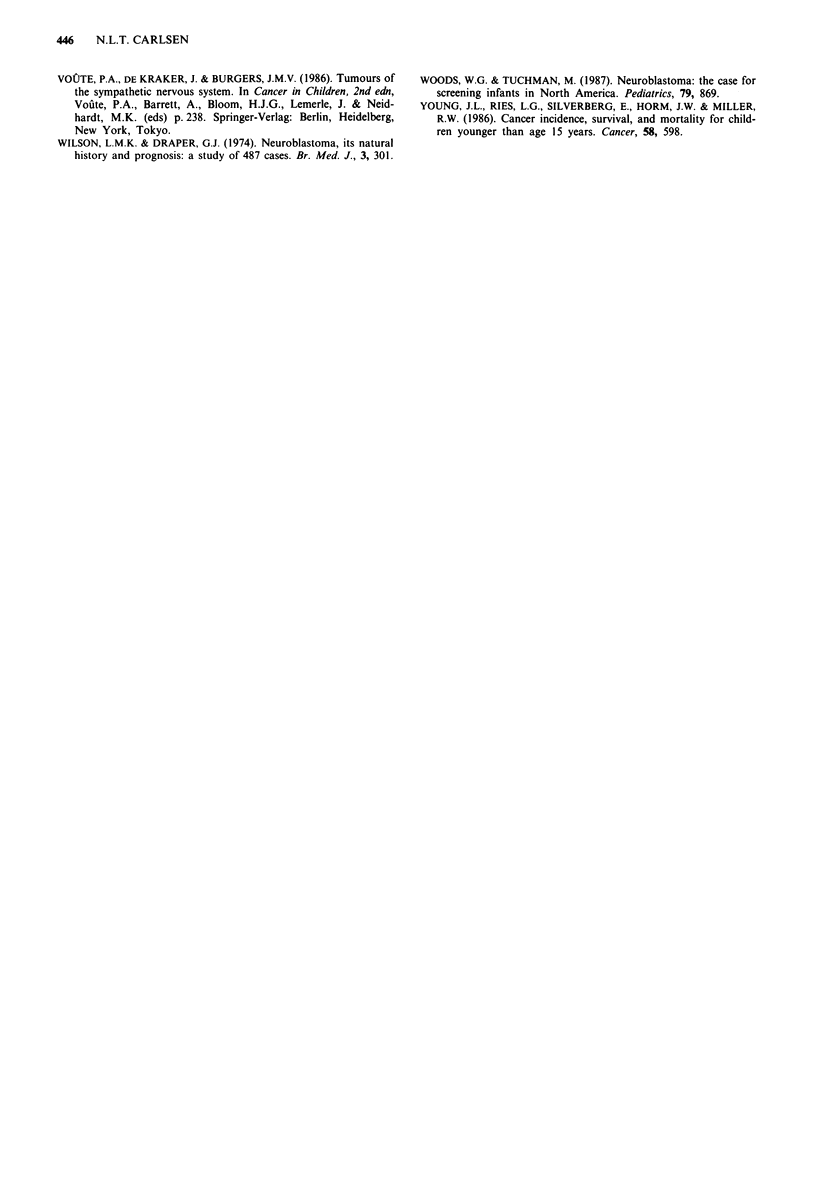# How frequent is spontaneous remission of neuroblastomas? Implications for screening.

**DOI:** 10.1038/bjc.1990.97

**Published:** 1990-03

**Authors:** N. L. Carlsen

**Affiliations:** Department of Paediatric Surgery, State University Hospital, Rigshospitalet, Copenhagen, Denmark.

## Abstract

The 'true' incidence of spontaneous regression of neuroblastomas is uncertain. However, the frequency of spontaneous regression is important when the benefits of screening procedures are considered. In the population-based Danish neuroblastoma survey 1943-80, spontaneous regression was documented in less than 2% of cases. However, the 'true' incidence may be higher. The epidemiological findings of increased incidence and survival rates with an unchanged mortality rate may suggest the inclusion of borderline lesions among 'truly' malignant neuroblastomas in recent decades in Denmark. However, it is more likely to be a result of improved diagnosis, changes in the social composition of the population and possibly unidentified environmental agents. However, if some premalignant lesions in fact had been included, they are most likely to be stages I-II tumours of infancy. In this study we describe cases of spontaneous regression of neuroblastoma from the Danish population-based survey 1943-80.


Br. J. Cancer (1990), 61, 441-446 © Macmillan Press Ltd., 1990

### How frequent is spontaneous remission of neuroblastomas? Implications for screening

N.L.T. Carlsen

*Department of Paediatric Surgery, State University Hospital, Rigshospitalet, 9 Blegdamsvej, DK-2100 Copenhagen Ø, Denmark.*

**Summary** The 'true' incidence of spontaneous regression of neuroblastomas is uncertain. However, the frequency of spontaneous regression is important when the benefits of screening procedures are considered. In the population-based Danish neuroblastoma survey 1943-80, spontaneous regression was documented in less than 2% of cases. However, the 'true' incidence may be higher. The epidemiological findings of increased incidence and survival rates with an unchanged mortality rate may suggest the inclusion of borderline lesions among 'truly' malignant neuroblastomas in recent decades in Denmark. However, it is more likely to be a result of improved diagnosis, changes in the social composition of the population and possibly unidentified environmental agents. However, if some premalignant lesions in fact had been included, they are most likely to be stages I-II tumours of infancy. In this study we describe cases of spontaneous regression of neuroblastoma from the Danish population-based survey 1943-80.

Numerous anecdotal reports of cases of spontaneous remission of neuroblastomas have been published. For example, a Danish patient diagnosed in 1941 has been the subject of several reports (Hansen, 1953; Rosendal, 1942; Visfeldt, 1963). Neuroblastomas undergoing spontaneous regression form 17% of the cases of spontaneous remission in man collected from the literature by Everson and Cole (1966). In a review of cases registered in Children's Cancer Study Group, Evans *et al.* (1976) estimated the frequency to be 8% of cases (including stage IV-S cases). Pritchard and Kemshead (1983), excluding stage IV-S cases, found the frequency to be only 1-2% in their experience. However, a population-based study has not been carried out so far.

Most childhood neuroblastomas are likely to be congenital (Birch *et al.*, 1980; Carlsen, 1988*a*; Carlsen *et al.*, 1986*a*; Rubin, 1968; Sutow, 1958; Wilson & Draper, 1974), and the prognosis is poor for most patients over the age of 2 years. However, the benefits of a mass screening in infants (Sawada *et al.*, 1984*a*, 1987*a*; Scriver *et al.*, 1987; Woods & Tuchman, 1987) has been questioned because it is argued that some cases detected at screening might have subsequently regressed spontaneously (Norman *et al.*, 1987; Pastore *et al.*, 1984).

The Danish population-based survey 1943-80 (Carlsen, 1986, 1988*b*; Carlsen *et al.*, 1986*a*, *b*, 1987) offers a unique opportunity to estimate the frequency of spontaneous regression from clinical overt disease in an unselected population of neuroblastomas of childhood. This study reports and discusses cases of confirmed regression and also the questionable cases. The study also considers age, stage and fate of cases found incidentally by abdominal examination or chest X-rays, as these cases are suggested to be equivalent to the cases found by screening (Kosloske *et al.*, 1987; Sawada *et al.*, 1988). As data from the Danish survey indicate a zero-time shift (Bailar & Smith, 1986; Feinstein *et al.*, 1985) in the study period (Carlsen, 1986; Carlsen *et al.*, 1986*a*, 1987), the material was re-examined for epidemiological data suggestive of the inclusion of premalignant lesions among the pool of 'real' neuroblastomas.

### Patient population

The patient population consisted of all the 250 cases of childhood neuroblastoma in Denmark from 1943 to 1980 (excluding five patients who were resident outside Denmark when diagnosed) (Carlsen, 1986, 1988*b*; Carlsen *et al.*, 1986*a*, 1987). The tumours were staged according to the system of Evans *et al.* (1971) as previously reported (Carlsen *et al.*, 1986*b*). Neuroblastoma *in situ* (Beckwith & Perrin, 1963; Ikeda *et al.*, 1981; Turkel & Itabashi, 1974), primary intracerebral neuroblastomas and neuroepitheliomas of the peripheral nerves were not included in the material. The hospital records of all but two children were studied (one record lost, one never hospitalised), and cases in which the tumour showed possible signs of spontaneous regression were selected for case reports with comments. Two of the five case histories have previously been reported (Carlsen & Nielsen, 1980; Rechnitzer & Hansen, 1980). All the 10 stage IV-S cases in the study period and also all 20 cases found incidentally were selected for this study (Carlsen *et al.*, 1986*a*, 1987), and the secular trends of frequency of these patients and also the trends in age and stage distribution were considered.

### Case histories and discussion

### *Spontaneous regression from clinical overt disease*

*Case history no. 1* An 8-month-old boy was referred to hospital in 1958 due to paraplegia. Intravenous pyelogram showed a mass under the left kidney. No other lesions were detectable, and in retrospect the tumour is assigned to stage II of Evans *et al.* (1971). Only biopsy was performed, which revealed an undifferentiated tumour suggestive of a neuroblastoma (the histological material could not be re-examined). No treatment was given apart from massive doses of vitamin $B_{12}$ for several years (Bodian, 1959). The tumour gradually disappeared and the paraplegia improved over a period of 10 months. VMA excretion was normal in 1964. The patient has been well for more than 20 years with only slight neurological sequelae.

The histological diagnosis is uncertain.

*Case history no. 2* A 3-week-old boy was admitted to hospital in 1967 due to paraplegia. A mass above the right kidney was seen on intravenous pyelogram. No other lesions were detectable, and in retrospect the tumour is assigned to stage II of Evans *et al.* (1971). VMA excretion was abnormally high. Only biopsy was done revealing an undifferentiated neuroblastoma. During radiotherapy consisting of 29 Gy the tumour disappeared completely and the paraplegia improved, but VMA excretion increased and skin metastases developed. No further therapy was given. Multiple metastases of skin, bone and distant lymph nodes developed within the next 2 years. They remain unchanged for 3 years, after which some spontaneously regressed while others remained stable. Biopsy of skin and bone metastases taken 8 and 12 years after diagnosis showed now ganglioneuroma, and the patient, although with neurological sequelae, has remained otherwise

Received 8 November 1988; and in revised form 18 September 1989.

asymptomatic for more than 15 years. VMA excretion is still abnormally high (Rechnitzer & Hansen, 1980).

*Case history no. 3* A 13-month-old boy was referred to hospital in 1961 due to a common cold. A mass was found on abdominal examination, and an intravenous pyelogram showed a tumour above the left kidney. No other lesions were detectable, and the tumour is assigned to stage II of Evans *et al.* (1971) in retrospect. Only biopsy was done, which revealed an undifferentiated neuroblastoma. Radiotherapy was given consisting of 25 Gy and the tumour diminished in size and was totally resected 1 month later by a secondary operation. Histological examination of the tumour revealed now only mature ganglioneuroma tissue. VMA was normal in 1966. The patient has been well for more than 20 years (Carlsen & Nielsen, 1980).

The histological maturation following irradiation may simply represent undifferentiated neuroblastoma-cell kill with subsequent overgrowth of the mature ganglioneuroma elements in the tumour (Cheson *et al.*, 1986).

*Case history no. 4* A 10-month-old girl was referred to hospital in 1968 due to paraplegia and bladder disturbances. A lumbar myelogram showed a lumbar extradural block. An extradural tumour with extensions in the intervertebral foramina was resected by laminectomy. The histological diagnosis was neuroblastoma. VMA was normal after surgery. No residual extraspinal disease could be detected by radiographical examination or by abdominal exploration 2 months later. No other lesions were detectable, and the tumour is in retrospect assigned to stage II of Evans *et al.* (1971). No further therapy was given and the patient has been well for more than 15 years.

Beckwith and Martin (1968) have suggested that some dumb-bell tumours might originate from dorsal root ganglia, and neuroblastomas derived from this source might be non-secretors of catecholamines; hence, the extraspinal component of the tumour might have been very small.

Others have also observed that children with dumb-bell primary neuroblastomas may be long-term survivors following total or subtotal resection of the intraspinal, extradural component of the mass without further treatment of the residual extraspinal disease (Pritchard & Kemshead, 1983).

*Case history no. 5* A 10-month-old girl was referred to hospital in 1975 due to periorbital ecchymoses and irritability. She had stage IV disease with multiple metastases of bones, including orbital bones, distant lymph nodes and a large abdominal mass. VMA excretion was grossly elevated. No treatment was given initially. There was no progression of the disease during the next 5 months. Therefore treatment was initiated with a combination of pulsed cyclophosphamide and vincristine at weekly intervals. The tumour responded to treatment, and 3 months later the primary tumour was totally resected. No irradiation was given. The bone lesions continued to heal, and the patient achieved a complete remission after 53 weeks of treatment. The treatment was then discontinued, and the patient has been well for more than 10 years.

The usual prolonged arrest of tumour growth may suggest that some unknown 'regression mechanisms' probably play a part in the complete response to treatment.

Thus, during the 38-year period covered by this study only two cases with spontaneous regression were seen; another three cases were observed in which some of the unusual course of the disease could be attributed to 'spontaneous regression mechanisms'. Together, these cases seen in 250 Danish children with neuroblastoma give an optimistic frequency of 2%. The cases, distributed by decade of diagnosis, are shown in Table I. It is seen that the frequency ranges from 0 to 4% of cases during the four decades. It is noticeable that all five patients were less than 14 months old when diagnosed. Four had stage II disease, one of whom progressed to stage IV before spontaneous regression took place, and one had stage IV disease at diagnosis. The latter received potentially curative treatment.

Ten stage IV-S cases were diagnosed during the study period. One was 14 months old at diagnosis, the other nine were less than 6 months old. Six died either as a result of the augmented intra-abdominal pressure from the expanding liver or from lung complications. All four survivors received treatment (Carlsen *et al.*, 1986*a*). However, as they probably all might have recovered without treatment if the complications had been avoided or treated more appropriately (Evans *et al.*, 1981), all 10 patients are shown in Table I distributed by decade of diagnosis. Patients with stage IV-S form 4% of cases.

### *Incidentally discovered tumours*

Tumours were found incidentally in 20 patients (Carlsen *et al.*, 1987) either by abdominal examination (11 patients) or by a routine chest X-ray (nine patients) (Table I). Ten patients had stage I disease; all have survived except one who died from progressive disease (an infant with a sacrooccygeal teratoma with areas of neuroblastoma among other structures (Carlsen *et al.*, 1986*a*)). Eight had stage II disease; all survived except one who died from surgical complications. Both patients with stage III disease succumbed, one from surgical complications, the other from progressive disease. Only six patients were older than 3 years at diagnosis, of whom four (all older than 5 years) had intrathoracic tumours (Table II). Even if it is likely that only the tumours in the 14

**Table I** Trends in the distribution of 'probable spontaneous regressing' neuroblastomas in Denmark 1943–80

| | *1943–49* | *1950–59* | *1960–69* | *1970–80* | *1943–80* |
|---|---|---|---|---|---|
| 1. Cases with unquestional spontaneous regression | 0 | 1 | 1 | 0 | 2 |
| 2. Cases with probable spontaneous regression | 0 | 0 | 2* | 1 | 3* |
| 3. Cases with stage IV-S disease (including 6 fatalities) | 1(1) | 1(1) | 6(3) | 2(1) | 10(6) |
| 4. Cases found incidentally (including 4 fatalities) | | | | | |
| a. Abdominal mass | 0 | 0 | 2(1)* | 9(2) | 11(3)* |
| b. Chest X-ray | 0 | 2(1) | 1 | 6 | 9(1) |
| 5. Cases found incidentally less than 3 years old (including 3 fatalities) | | | | | |
| a. Abdominal mass | 0 | 0 | 2(1)* | 7(2) | 9(3)* |
| b. Chest X-ray | 0 | 0 | 1 | 4 | 5 |
| Total number of neuroblastoma cases in each decade | 27 | 48 | 69 | 106 | 250 |
| Total number of long-term survivors in each decade | 0 | 4 | 14 | 35 | 53 |
| Survivors found incidentally (4) | 0 | 1 | 2 | 13 | 16 |
| Survivors from 1 + 2 + 3 + 5 | 0 | 1 | 7 | 11 | 19 |
| Percent of long-term survivors in each decade | 0% | 8% | 20% | 33% | 21% |
| a. Found incidentally (4) | 0% | 2% | 3% | 12% | 6% |
| b. From 1 + 2 + 3 + 5 | 0% | 2% | 10% | 10% | 8% |

*One patient with a probable spontaneous regression tumor was found incidentally. Numbers in parentheses indicate fatalities. Ten of a total of 19 stage I patients 1943–80 were found incidentally (53%); 8 of a total of 48 stage II patients were found incidentally (17%); 2 of a total of 34 stage III patients were found incidentally (6%).

**Table II** Age, stage and survival according to clinical or incidental presentation

| *Age at diagnosis* | *Stage I* | *Stage II* | *Stage III* | *Stage IV* | *Stage IV-S* | *Unknown* | *Total* |
|---|---|---|---|---|---|---|---|
| 0–5 months | | | | | | | |
| clinically | 1/2 | 6/6 | 1/2 | 0/9 | 3/9 | 0/1 | 11/29 |
| incidentally | 0/1 | 1/1 | – | – | – | – | 1/2 |
| 6–11 months | | | | | | | |
| clinically | 2/3 | 6/9 | 0/2 | 2/14 | – | – | 10/28 |
| incidentally | 2/2 | 2/3 | – | – | – | – | 4/5 |
| 12–23 months | | | | | | | |
| with symptoms occurring in the first year of life | 0/1 | 0/2 | 0/1 | 0/4* | 1/1 | – | 1/9 |
| other clinically | 1/1 | 2/5 | 1/9 | 1/20 | – | – | 5/35 |
| incidentally | 2/2 | 4/4 | – | – | – | – | 6/6 |
| 24–35 months | | | | | | | |
| clinically | – | 1/2 | 0/5 | 0/25* | – | – | 1/32 |
| incidentally | – | – | 0/1 | – | – | – | 0/1 |
| 36–47 months | | | | | | | |
| clinically | 2/2 | 2/3 | 0/3 | 0/20 | – | – | 4/28 |
| incidentally | 1/1 | – | – | – | – | – | 1/1 |
| 48–59 months | | | | | | | |
| clinically | – | 0/2 | 0/2 | 0/9 | – | – | 0/13 |
| incidentally | – | – | – | – | – | – | – |
| ⩾60 months | | | | | | | |
| clinically | – | 5/11 | 0/8 | 0/36* | – | 0/1 | 5/56 |
| incidentally | 4/4 | – | 0/1 | – | – | – | 4/5 |
| *Total* | | | | | | | |
| clinically | 6/9 | 22/40 | 2/32 | 3/137 | 4/10 | 0/2 | 37/230 |
| incidentally | 9/10 | 7/8 | 0/2 | – | – | – | 16/20 |

*Six patients with stage IV disease at diagnosis had undeniable symptoms during the first year of life.

patients below 3 years of age could possibly have been found by screening in infancy (Sawada *et al.*, 1987*a*; Scriver *et al.*, 1987; Woods & Tuchman, 1987), all 20 patients are shown in Table I distributed by decade of diagnosis, and the 14 cases under 3 years of age separately. Incidental cases form only 8% of cases, but it is seen from Table I that the frequency increased from 0 to 14% from 1943 to 1980 and, furthermore, 30% of long-term survivors 1943–80 were found incidentally. Even if it remains a matter of speculation as to whether some stages I–II infants diagnosed incidentally or by screening would have regressed spontaneously (as they almost always receive potentially curative treatment (Evans *et al.*, 1976)), the major criticism against mass screening is based on this postulate (Norman *et al.*, 1987; Pastore *et al.*, 1984).

If in speculating about the proportion of cases that might have regressed spontaneously, the cases under 2 years with stages I–II, who did not relapse and were found incidentally, are added to the probable or confirmed spontaneous regression cases and the 10 with stage IV-S, a combined frequency will be obtained of 26/250 = 10% of cases 'who might probably have regressed spontaneously' in Denmark 1943–80. However, this estimate of the maximum spontaneous remission rate is highly speculative and optimistic. Long-term survivors from this speculative group form 36% of all long-term survivors during the study period.

The substances of Tables II and III have been published before (Carlsen *et al.*, 1986*a*, 1987), and the tables are only added to help in understanding the issue of a possible lead-time bias, and the speculation about the proportion of cases that might have their prognosis influenced by a screening programme.

### Questions addressed by these data

*Do epidemiological data suggest the inclusion of premalignant lesions among neuroblastoma cases?*

The incidence of neuroblastoma increased in Denmark during 1943–80 to a level corresponding to that in the USA (Young *et al.*, 1986). The increase relates solely to children under 5 years of age, and is most pronounced in infants under 1 year. On the other hand, the mortality rate has not changed significantly (Carlsen, 1986). During the same period the long-term survival has gradually improved due to a

**Table III** Trends in the distribution of stages and ages at diagnosis in Denmark 1943–80

| *Survivors/total* | *1943–49* | *1950–59* | *1960–69* | *1970–80* | *1943–80* |
|---|---|---|---|---|---|
| Below 1 year of age | | | | | |
| Stage I | 0/0 | 0/0 | 0/0 | 5/8 | 5/8 |
| Stage II | 0/1 | 2/2 | 5/8 | 8/8 | 15/19 |
| Stage III | 0/0 | 0/1 | 0/0 | 1/3 | 1/4 |
| Stage IV | 0/2 | 0/5 | 1/6 | 1/10 | 2/23 |
| Stage IV-S | 0/1 | 0/1 | 2/5 | 1/2 | 3/9 |
| Unknown | 0/0 | 0/1 | 0/0 | 0/0 | 0/1 |
| Total below 1 year of age | 0/4 | 2/10 | 8/19 | 16/31 | 26/64 |
| 1 year of age or older | | | | | |
| Stage I | 0/0 | 2/2 | 2/3 | 6/6 | 10/11 |
| Stage II | 0/5 | 0/1 | 3/8 | 11/15 | 14/29 |
| Stage III | 0/4 | 0/9 | 0/6 | 1/11 | 1/30 |
| Stage IV | 0/14 | 0/25 | 0/32 | 1/43 | 1/114 |
| Stage IV-S | 0/0 | 0/0 | 1/1 | 0/0 | 1/1 |
| Unknown | 0/0 | 0/1 | 0/0 | 0/0 | 0/1 |
| Total 1 year of age or older | 0/23 | 2/38 | 6/50 | 19/75 | 27/186 |
| Per cent 1 year of age or older | 85% | 79% | 72% | 71% | 74% |

combination of a higher frequency of lower stages of the disease (I–II), younger ages and multimodal treatment including chemotherapy (Carlsen *et al.*, 1986*a*). Thus, a striking result from the Danish survey is the clear demonstration of a zero-time shift (Table III). As the survivor have been followed for at least 7 years and are considered cured, the upward survival trend is not a simple lead-time bias, i.e. there is earlier diagnosis but death from neuroblastoma still occurs (Bailar & Smith, 1986; Feinstein *et al.*, 1985). It is well recognised that a shift in diagnostic criteria to include lesions that have the microscopical appearance of cancer but not its biological behaviour among truly malignant neoplasms will result in higher incidence rates, lower stages at diagnosis and improved survival rates, but with unchanged mortality rates (Bailar & Smith, 1986). Thus, the data may be suggestive of the inclusion of a proportion of 'benign' or borderline lesions in the recent decades. The number of cases in the highly speculative group of 'probably spontaneous regressing tumours' cannot explain the rise in incidence on their own (Table I), and several more plausible explanations for the rise can be given (Carlsen, 1986, 1988*b*). However, the suggestion that neuroblastomas lacking malignant behaviour are included in the pool of 'real' neuroblastomas must be considered seriously, as the implications are substantial. It is striking that the prognosis was favourable for stage I disease in all ages and stage II disease of infancy during the whole period (Table III).

*Are some stages I–II tumours of infancy premalignant lesions?*

There are limited data on the proportion of stage I tumours with evidence of malignant behaviour, as they nearly always receive potentially curative treatment (Evans *et al.*, 1976). All 19 stage I patients in Denmark underwent complete tumour resection with only one relapse (Carlsen *et al.*, 1986*a*). In contrast, all stage II tumours of Evans *et al.* (1971) have demonstrated local invasiveness, and some the ability to metastasise to local lymph nodes. Nineteen patients with stage II disease were under one year old (Tables II and III) and all but two received potentially curative treatment. Four died but only two from documented progressive disease; two others had evidence of tumour progression, one of whom recovered spontaneously (case history 2), the other following treatment with chemotherapy. Another infant recovered spontaneously (case history 1). Thus, apart from local invasiveness some stage II tumours of infancy have demonstrated truly malignant behaviour. However, a similar proportion showed spontaneous regression. Infants in stages II and IV-S provided the majority of documented spontaneous regression in other studies (Bodian, 1959; Evans *et al.*, 1976; Everson & Cole, 1966; Gross *et al.*, 1959). It would be in accordance with Foulds' description of the stepwise evolution of tumours from a (benign) proliferative lesion to an increasing autonomous and malignant neoplasm (Klein & Klein, 1986; Knudson & Meadows, 1980; Moolgavkar & Knudson, 1981; Nicolson, 1987) to suggest that some congenital neuroblastomas are dependent on external growth factors during the first few years of life (Carlsen, 1988*a*), a period of proliferation, differentiation and involution of the paraganglia system (Voûte *et al.*, 1986).

*Rationale for screening*

Much evidence is compatible with the suggestion that most neuroblastomas are congenital (Birch *et al.*, 1980; Carlsen, 1988*a*; Carlsen *et al.*, 1986*a*; Rubin, 1968; Sutow, 1958; Wilson & Draper, 1974), and the age with stage distribution strongly suggests that disseminated cases have passed through lower undetected stages at younger ages before they are diagnosised (Carlsen, 1988*a*). The disease is notorious for its vagueness of symptoms (Carlsen *et al.*, 1987; Wilson & Draper, 1974) and, as age under one year and stages I–II disease have a crucial effect on prognosis, paediatric oncologists emphasise the importance of a good abdominal examination whenever a child is seen by a physician, and mass screening programmes in infants have been proposed (Sawada *et al.*, 1984*a*, 1987*a*; Scriver *et al.*, 1987; Woods & Tuchman, 1987). Table II shows the number of neuroblastoma patients in Denmark found at each age, either clinically or incidentally. One can speculate as to whether the prognosis of all patients with stages III–IV disease diagnosed between age 6 and 47 months (104/250 = 42%) could be influenced by screening at age 6 months, provided that 65% of all childhood neuroblastomas could be detected clinically or by screening before or at 6 months, as suggested from Japan (Sawada *et al.*, 1987*b*) (the cumulative percentage of cases reaches 65% between age 3 and 4 years in Denmark). The lead-time in this estimate is, however, considerably longer than suggested by Pastore *et al.* (1984). In this context it is noticeable that of 11 patients with undeniable signs of the disease in the first year of life (Carlsen, 1986), nine were diagnosed between age 12 and 23 months, one at 25 months and one at 94 months (Table II).

*What can we learn from the preliminary results of screening in Japan?*

In the recent birth cohorts in Denmark (Carlsen, 1986) and in the USA (Young *et al.*, 1986) 1 in 7,000 live births will develop neuroblastoma before 15 years of age. If 65% of all childhood neuroblastomas could be detected before or at the age of 6 months, as suggested in Japan (Sawada *et al.*, 1987*b*), then the expected prevalence by screening at age 6 months would be approximately 1 in 11,000 live births (65/100 × 1/7,000). However, the proof that screening can only detect cases that would have progressed to clinical disease depends on an appropriate fall in the incidence in older children and a decline in mortality rate (Carlsen, 1988*a*; Draper, 1988; Mauer, 1988). It is therefore of some concern that the incidence appears to increase with screening in Japan (Nishi *et al.*, 1987; Sawada, 1986; Sawada *et al.*, 1984*a*, *b*, *c*) so that now 1 in 5,000 is detected at 6 months of age (Nishi *et al.*, 1987; Sawada *et al.*, 1987*a*, *b*). This prevalence appears to be substantially higher than the cumulative birth cohort incidence rate under the age of 15 years in Denmark and the USA. The fact that screen-detected early stage tumours (Hayashi *et al.*, 1988; Kaneko *et al.*, 1987, 1988) are not believed to evolve into advanced stage tumours from a cytogenetic viewpoint, is of equal concern. Both studies of screen-detected tumours were missing the early stage tumours with near-diploidy or hypo-tetraploidy with structural abnormalities associated with older children and poor prognosis (Hayashi *et al.*, 1988; Kaneko *et al.*, 1987).

What are we dealing with? Is a substantial proportion of the screen-detected cases in Japan spontaneous regressing tumours, or not malignancies at all? Neuroblastoma *in situ* (Beckwith & Perrin, 1963, Ikeda *et al.*, 1981; Turkel & Itabashi, 1974), which most likely is a normal variation in the morphogenesis of the adrenal gland, has been found by random autopsy studies in infants under 3 months old in incidences of 1 in 179 to 1 in 259 (Beckwith & Perrin, 1963). Could some screen-detected cases virtually be neuroblastoma *in situ* cases?

### Tentative conclusions

The prognosis remains poor for most patients with neuroblastoma who are diagnosed at unfavourable ages and stages due to the vagueness of symptoms. Therefore, screening for the disease is desirable.

Most data concerning the natural history of this tumour are compatible with the suggestion that they are often congenital. Therefore, screening should be done as soon after birth as possible. However, the proof of the hypothesis depends on the finding that a higher frequency of cases found in infancy by screening will eventually be followed by an appropriate fall in the number of cases diagnosed at older ages and a decline in mortality rate.

The occasional observation of spontaneous regression of tumours in infants and the rare observation of the same phenomenon in older children is compatible with the concept that tumours develop by a series of changes from a dependent to an increasingly autonomous and malignant neoplasm.

Insufficient data exist concerning the frequency of stages I–II tumours of infancy that are dependent tumours, which might regress spontaneously. Therefore, stages I–II cases of infancy should be treated cautiously with the possibility of regression in mind.

Screening studies should not be undertaken unless there exists a reliable incidence and mortality rate for the population to allow close observation of the epidemiological rates. Furthermore, cases found by screening should be intensively studied for karyotypic pattern, oncogene expression and cellular DNA content, among others.

I am indepted to Drs P.V. Bro, G. Erichsen, B. Hamborg-Pedersen, U. Hesselbjerg and H. Schroeder for the collection of this patient population.

### References

BAILAR, J.C. III & SMITH, E.M. (1986). Progress against cancer? *N. Engl. J. Med.*, **314**, 126.

BECKWITH, J.B. & MARTIN, R.F. (1968). Observations of the histopathology of neuroblastoma. *J. Pediatr. Surg.*, **3**, 106.

BECKWITH, J.B. & PERRIN, E.V. (1963). In situ neuroblastoma: a contribution to the natural history of neural crest tumors. *Am. J. Pathol.*, **43**, 1089.

BIRCH, J.M., MARSDEN, H.B. & SWINDELL, R. (1980). Incidence of malignant disease in childhood: a 24-year review of the Manchester Children's Tumour Registry data. *Br. J. Cancer*, **42**, 215.

BODIAN, M. (1959). Neuroblastoma. *Pediatr. Clin. North Am.*, **6**, 449.

CARLSEN, N.L.T. (1986). Epidemiological investigations on neuroblastomas in Denmark 1943–1980. *Br. J. Cancer*, **54**, 977.

CARLSEN, N.L.T. (1988*a*). Annotation. Why age has independent prognostic significance in neuroblastomas. Evidence for intrauterine development, and implications for the treatment of the disease. *Anticancer Res.*, **8**, 255.

CARLSEN, N.L.T. (1988*b*). Regional differences in incidence of neuroblastoma in Denmark in 1943–1980. *Ugeskr. Læger.*, **150**, 2511.

CARLSEN, N.L.T., CHRISTENSEN, I.J., SCHROEDER, H. & 5 others (1986*a*). Prognostic factors in neuroblastomas treated in Denmark from 1943 to 1980. A statistical estimate of prognosis based on 253 cases. *Cancer*, **58**, 2726.

CARLSEN, N.L.T., CHRISTENSEN, I.J., SCHROEDER, H. & 4 others (1986*b*). Prognostic value of different staging systems in neuroblastomas and completeness of tumour excision. *Arch. Dis. Child.*, **61**, 832.

CARLSEN, N.L.T. & NIELSEN, O.H. (1980). Abdominal neuroblastomas. An analysis of 48 cases. *Ugeskr. Læger.*, **142**, 2972.

CARLSEN, N.L.T., SCHROEDER, H., CHRISTENSEN, I.J. & 4 others (1987). Signs, symptoms, metastatic spread and metabolic behaviour of neuroblastomas treated in Denmark during the period 1943–1980. *Anticancer Res.*, **7**, 465.

CHESON, B.D., JASPERSE, D.M., CHUN, H.G. & FRIEDMAN, M.A. (1986). Differentiating agents in the treatment of human malignancies. *Cancer Treat. Rev.*, **13**, 129.

DRAPER, G.J. (1988). Screening for neuroblastoma. *Br. Med. J.*, **297**, 152.

EVANS, A.E., BAUM, E. & CHARD, R. (1981). Do infants with stage IV-S neuroblastoma need treatment? *Arch. Dis. Child.*, **56**, 271.

EVANS, A.E., D'ANGIO, G.J. & RANDOLPH, J. (1971). A proposed staging for children with neuroblastoma. *Cancer*, **27**, 374.

EVANS, A.E., GERSON, J. & SCHNAUFER, L. (1976). Spontaneous regression of neuroblastoma. *Natl Cancer Inst. Monogr.*, **44**, 49.

EVERSON, T.C. & COLE, W.H. (1966). *Spontaneous Regressing of Cancer*. W.B. Saunders: Philadelphia.

FEINSTEIN, A.R., SOSIN, D.M. & WELLS, C.K. (1985). The Will Rogers phenomenon. Stage migration and new diagnostic techniques as a source of misleading statistics for survival in cancer. *N. Engl. J. Med.*, **312**, 1604.

GROSS, R.E., FARBER, S. & MARTIN, L.W. (1959). Neuroblastoma sympatheticum. A study and report of 217 cases. *Pediatrics*, **23**, 1179.

HANSEN, P.B. (1953). Sympathicoblastoma of the adrenal medulla with osseous metastases. *Acta Radiol.*, **40**, 500.

HAYASHI, Y., INABA, T., HANADA, R. & YAMAMOTO, K. (1988). Chromosome findings and prognosis in 15 patients with neuroblastoma found by VMA mass screening. *J. Pediatr.*, **112**, 567.

IKEDA, Y., LISTER, J., BOUTON, J.M. & BUYUKPAMUKCU, M. (1981). Congenital neuroblastoma, neuroblastoma *in situ*, and the normal fetal development of the adrenal. *J. Pediatr. Surg.*, **16**, 636.

KANEKO, Y., KANDA, N., MASEKI, N. & 5 others (1987). Different karyotypic patterns in early and advanced stage neuroblastomas. *Cancer Res.*, **47**, 311.

KANEKO, Y., MASEKI, N., SAKURAI, M. & 4 others (1988). Chromosomes and screening for neuroblastoma. *Lancet*, **i**, 174.

KLEIN, G. & KLEIN, E. (1986). Conditioned tumorigenicity of activated oncogenes. *Cancer Res.*, **46**, 3211.

KNUDSON, A.G. & MEADOWS, A.T. (1980). Regression of neuroblastoma IV-S: a genetic hypothesis. *N. Engl. J. Med.*, **302**, 1254.

KOSLOSKE, A.M., BHATTACHARYYA, N. & DUNCAN, M.H. (1987). 'Incidental' neuroblastoma. *Lancet*, **ii**, 565.

MAUER, A.M. (1988). Screening for neuroblastoma. *J. Pediatr.*, **112**, 576.

MOOLGAVKAR, S.H. & KNUDSON, A.G. (1981). Mutation and cancer: a model for human carcinogenesis. *J. Natl Cancer Inst.*, **66**, 1037.

NICOLSON, G.L. (1987). Tumor cell instability, diversification and progression to the metastatic phenotype: From oncogene to oncofetal expression. *Cancer Res.*, **47**, 1473.

NISHI, M., MIYAKE, H., TAKEDA, T. & 4 others (1987). Effects of the mass screening of neuroblastoma in Sapporo City. *Cancer*, **60**, 433.

NORMAN, M.G., DIMMICK, J.E. & TEASDALE, M. (1987). Screening for neuroblastoma. *Lancet*, **ii**, 565.

PASTORE, G., MERLETTI, F. & MAGNANI, C. (1984). Screening for neuroblastoma. *Lancet*, **ii**, 752.

PRITCHARD, J. & KEMSHEAD, J. (1983). Neuroblastoma: recent developments in assessment and mangement. In *Paediatric Oncology*, Duncan, E.W. (ed.) p. 69. Springer-Verlag: Heidelberg.

RECHNITZER, C. & HANSEN, S.W. (1980). Maturation of neuroblastoma. Long-term survival with disseminated osseous metastases. *Ugeskr. Læger.*, **142**, 2976.

ROSENDAL, T. (1942). Two cases of sympathicoblastoma of the suprarenal gland with metastases to the cranium and the tubular bones. *Acta Radiol.*, **23**, 462.

RUBIN, P.R. (1968). Comment. Cure and the growth rate of childhood tumors. *JAMA*, **205**, 113.

SAWADA, T. (1986). Outcome of 25 neuroblastomas revealed by mass screening in Japan. *Lancet*, **i**, 377.

SAWADA, T., HIRAYAMA, M., NAKATA, T. & 10 others (1984*a*). Mass screening for neuroblastoma in infants in Japan. *Lancet*, **ii**, 271.

SAWADA, T., KAWAKATU, H., HORII, Y. & SUGIMOTO, T. (1988). Incidental neuroblastoma. *Lancet*, **i**, 364.

SAWADA, T., KAWAKATU, H. & SUGIMOTO, T. (1987*a*). Screening for neuroblastoma. *Lancet*, **ii**, 1204.

SAWADA, T., KIDOWAKI, T., SAKAMOTO, I. & 4 others (1984*b*). Neuroblastoma. Mass screening for early detection and its prognosis. *Cancer*, **53**, 2731.

SAWADA, T., KIDOWAKI, T., SUGIMOTO, T. & KUSUNOKI, T. (1984*c*). Incidence of neuroblastoma in infancy in Japan. *Med. Pediatr. Oncol.*, **12**, 101.

SAWADA, T., SUGIMOTO, T., TANAKA, T. & 4 others (1987*b*). Number and cure rate of neuroblastoma cases detected by the mass screening program in Japan: future aspects. *Med. Pediatr. Oncol.*, **15**, 14.

SCRIVER, C.R., GREGORY, D., BERNSTEIN, M. & 7 others (1987). Feasibility of chemical screening of urine for neuroblastoma case finding in infancy in Quebec. *Can. Med. Assoc. J.*, **136**, 952.

SUTOW, W.W. (1958). Prognosis in neuroblastoma of childhood. *Am. J. Dis. Child.*, **96**, 299.

TURKEL, S.B. & ITABASHI, H.H. (1974). The natural history of neuroblastic cells in the fetal adrenal gland. *Am. J. Pathol.*, **76**, 225.

VISFELDT, J. (1963). Transformation of sympathicoblastoma into ganglioneuroblastoma. *Acta Pathol. Microbiol. Scand.*, **58**, 414.

VOÛTE, P.A., DE KRAKER, J. & BURGERS, J.M.V. (1986). Tumours of the sympathetic nervous system. In *Cancer in Children, 2nd edn*, Voûte, P.A., Barrett, A., Bloom, H.J.G., Lemerle, J. & Neidhardt, M.K. (eds) p. 238. Springer-Verlag: Berlin, Heidelberg, New York, Tokyo.

WILSON, L.M.K. & DRAPER, G.J. (1974). Neuroblastoma, its natural history and prognosis: a study of 487 cases. *Br. Med. J.*, **3**, 301.

WOODS, W.G. & TUCHMAN, M. (1987). Neuroblastoma: the case for screening infants in North America. *Pediatrics*, **79**, 869.

YOUNG, J.L., RIES, L.G., SILVERBERG, E., HORM, J.W. & MILLER, R.W. (1986). Cancer incidence, survival, and mortality for children younger than age 15 years. *Cancer*, **58**, 598.